# Tuberculosis-Specific Antigen/Phytohemagglutinin Ratio Combined With GeneXpert MTB/RIF for Early Diagnosis of Spinal Tuberculosis: A Prospective Cohort Study

**DOI:** 10.3389/fcimb.2022.781315

**Published:** 2022-01-31

**Authors:** Yiwei Qi, Zhiwei Liu, Xiaojin Liu, Zhong Fang, Yanchao Liu, Feng Li

**Affiliations:** ^1^ Department of Neurosurgery, Tongji Hospital, Tongji Medical College, Huazhong University of Science and Technology, Wuhan, China; ^2^ Laboratory Sino-German Neuro-Oncology Molecular, Department of Neurosurgery, Tongji Hospital, Tongji Medical College, Huazhong University of Science and Technology, Wuhan, China; ^3^ Department of Orthopedic Surgery, Tongji Hospital, Tongji Medical College, Huazhong University of Science and Technology, Wuhan, China

**Keywords:** spinal tuberculosis, tuberculosis diagnosis, T-SPOT, GeneXpert MTB/RIF, spinal infection

## Abstract

Spinal tuberculosis (TB), the most common form of musculoskeletal tuberculosis, is an infection-related disease globally, with paraplegia occurring in severe cases. Therefore, identification of spinal TB at an early stage is important for early intervention and eventual therapy. In this study, we conducted a prospective cohort study in routine clinical practice to investigate the diagnosis of different TB tests. A total of 519 patients were recruited based on the radiology of spinal TB. The diagnostic model was computed by regression analysis and was determined by receiver operating characteristic (ROC) curve analysis. Specificity, sensitivity, predictive value, likelihood ratio, and accuracy were also computed and compared. GeneXpert MTB/RIF showed a higher positive rate compared to that in the acid-fast bacilli smear and *Mycobacterium* culture. The results also showed that the *Mycobacterium tuberculosis*-specific antigen/phytohemagglutinin ratio in the T-SPOT assay had a good performance in the preoperative diagnosis and prediction of spinal TB. The diagnostic model based on the ratio of tuberculosis-specific antigen/phytohemagglutinin combined with GeneXpert MTB/RIF showed better efficiency for spinal TB diagnosis. In summary, the tuberculosis-specific antigen/phytohemagglutinin ratio combined with GeneXpert MTB/RIF could provide an early diagnosis of spinal TB.

## Introduction

Tuberculosis (TB) is a kind of bacterial communicable disease caused by the *Mycobacterium tuberculosis* (MTB) complex (Furin et al., 2019). Spinal TB is a common spine infection disease, usually caused by lymphohematogenous spread from a pulmonary TB and occasionally by genitourinary system TB spread *via* venous or arterial routes in some cases (Ryu and Patel, 2015; Dunn and Ben Husien, 2018). Spinal TB is prevalent in children and young adults, usually during the initial latent phase of the infection (Ryu and Patel, 2015; Dunn and Ben Husien, 2018). Spinal TB is frequently asymptomatic in its early stage. Gradually, spinal TB can attribute to bone destruction, vertebral collapse, compression of the spinal cord and nerves, and eventually symptoms such as pain, numbness, and weakness (Yong et al., 2021). Therefore, early diagnosis of spinal TB is crucial for preventing the disease and improving therapeutic effects.

To date, spinal TB has been diagnosed by several approaches, including imaging, inflammatory parameters, bone biopsies for microbiological studies or histopathological studies, gene amplification, and immunoassay (Ryu and Patel, 2015; Dunn and Ben Husien, 2018). The various diagnostic methods of spinal TB have their advantages and disadvantages. The imaging presentation of spinal TB is highly consistent with other infections and spinal metastatic adenocarcinoma (Xiang et al., 2021). For inflammatory parameters, the elevation in C-reactive protein (CRP) and erythrocyte sedimentation rate (ESR) has indications but with relatively low specificity (Ryu and Patel, 2015). As the key element for acid-fast bacilli smear (AFBS) (Golden and Vikram, 2005), MTB culture (Dunn and Ben Husien, 2018), molecular polymerase chain reaction GeneXpert MTB/RIF (Chin et al., 2019), and histological exam (Dunn and Ben Husien, 2018), the tissue from bone biopsy has a higher diagnostic yield than pus. Therefore, the biopsy specimen should be as large as possible and include intact pieces of bone, shavings, and necrotic material (Ryu and Patel, 2015). Even so, biopsy-based tests are still less likely to yield positive results. Only 38% of cases were reported by AFBS (Dunn and Ben Husien, 2018), 50% of cases were identified through MTB culture (Lan et al., 2013), and 60% of patients were confirmed through histology from biopsy (Handa et al., 2010). GeneXpert MTB/RIF is a rapid test recommended by WHO with high specificity in the detection of extrapulmonary TB (EPTB) (World Health Organization, 2013) and similar sensitivity for the culture of spinal TB (Patel et al., 2020). Thus, negative microscopy screening for acid-fast bacillus, negative histopathology, negative GeneXpert MTB/RIF result, and failure of M. tuberculosis culture cannot exclude the diagnosis of spinal TB.

Hence, there is an urgent need to find more effective and objective methods for early spinal TB diagnosis. For immunoassay, T-SPOT is a commonly used test for interferon-γ (IFN-γ) release from blood, which is very useful for accurate detection of TB (Luo et al., 2020a). Usually, a high value of T-SPOT means a highly active TB (Janssens et al., 2007). T-SPOT has the potential to improve diagnostic capabilities before surgery. It was also found that MTB-specific antigen (TBAg) to phytohemagglutinin (PHA) ratio (TBAg/PHA ratio) in T-SPOT assay had better predicting capacity in TB (Wang et al., 2016). Therefore, we speculate that the use of combined diagnosis might better distinguish spinal TB from other spinal diseases.

In the present study, we investigated the possible clinical application of T-SPOT assay, GeneXpert MTB/RIF, AFBS, and other methods in the diagnosis of spinal TB. Our results collectively suggested that the TBAg/PHA ratio of T-SPOT assay combined with other methods could be used to establish a differential model for the differentiation of spinal TB from other spinal diseases. Thus, we can establish a new diagnostic model for early spinal TB diagnosis to improve clinical outcomes.

## Materials and Methods

### Study Design

**Figure 1:**
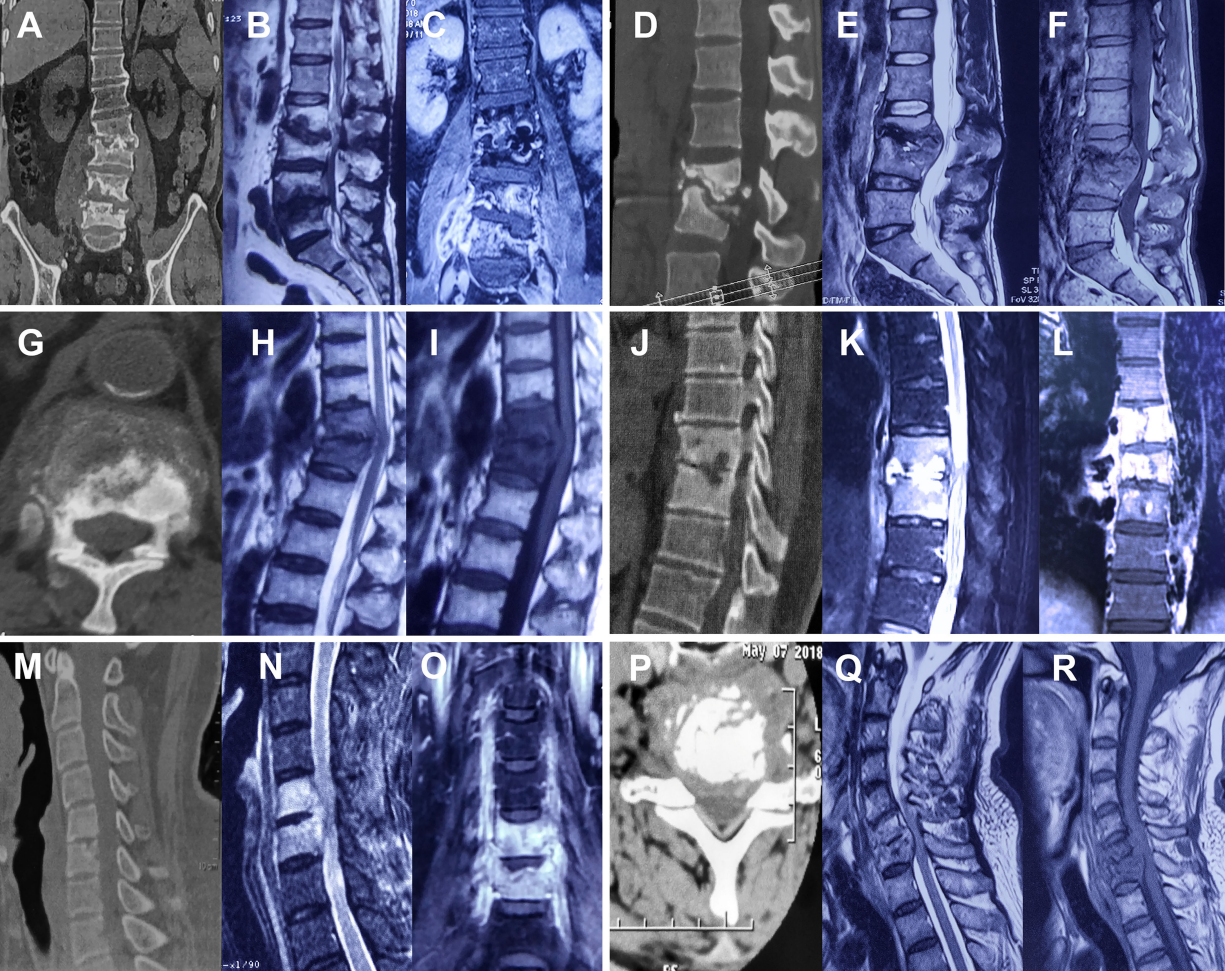
Typical spinal tuberculosis cases. Marginal erosions, bone destruction, soft tissue infection (abscesses), and spinal cord compression were shown on CT or MRI images. **(A–F)** Typical lumbar tuberculosis. **(G–L)** Typical thoracic tuberculosis. **(M–R)** Typical cervical tuberculosis.

A total of 519 patients were enrolled in this prospective cohort study at Tongji Hospital and Sino-French New City Hospital (a branch hospital of Tongji Hospital) from January 2018 to January 2021. All patients were recruited based on suspected spinal TB on radiology, including CT and MRI scans (**Figure 1**). The radiological inclusion criteria were briefly summarized as follows (Garg and Somvanshi, 2011): (1) irregular lytic lesions or bone destruction involving the vertebral body, endplate, or paradiscal margins, including fragmentary destruction, osteolytic destruction, and localized destruction with sclerotic margins; (2) disk destruction or narrowing of the intervertebral disk height; (3) calcifications formation or bone fragments with abscess; (4) collapse of vertebrae or kyphotic deformity; and (5) soft tissue involvement and large paravertebral abscess. Exclusion criteria included (1) undergoing anti-TB treatment for >2 weeks, (2) active malignancy undergoing therapy, (3) autoimmune disease, (4) pulmonary TB or other TB, (5) HIV infection, (6) refusal to sign informed consent, (7) one of the TB diagnostic tests was missed, (8) refusal of surgery.

Microscopy screening for AFBS, T-SPOT, GeneXpert MTB/RIF, and *Mycobacterium* TB culture for all patients was carried out by skilled laboratory physicians in the Department of Laboratory Medicine. TB histopathology was confirmed by the Pathology Department of Tongji Hospital, which showed caseous necrosis, Langhans-type giant cells, epithelioid histiocytes, and lymphocytes infiltration. The diagnosis of spinal TB was based on the following criteria according to WHO guidelines: (1) a positive culture for spinal TB and (2) typical histopathology for TB. The study was approved by the Ethics Committee of Tongji Hospital, Tongji Medical College, Huazhong University of Science and Technology. Written informed consent was obtained from all participants.

### Tissue Sample Acquiring and TB Tests

All tissues samples were collected through various biopsy methods, including fluoroscopy-guided percutaneous biopsy and open biopsy during surgical procedures. A STERYLAB S.R.L biopsy needle (9 G diameter, 15 cm in length) was used for fluoroscopy-guided percutaneous biopsy. Intact pieces of bone tissue were taken for further examination, including microscopy screening for AFBS, GeneXpert MTB/RIF, histopathology, and MTB culture.

### Tissue Prepared for Acid-Fast Bacillus or Tuberculosis Culture

Tissues were prepared as the previous reports (Feyzioglu et al., 2014) and under the guidance of BD BACTEC™ MGIT™ 960 system protocols. Five milliliters of Middlebrook 7H9 broth base and 1 g n-acetyl-l-cysteine (NALC) were added to the tissue samples, grounded into slurry with a grinder. After the slurry was treated with an equal volume of 2% NALC-NaOH in a 50-ml centrifuge tube, the slurry was vortexed for 20 s and incubated at room temperature for 15 min. Then, the incubated mixture was diluted to 50 ml with phosphate-buffered saline (PBS, pH = 6.8) and centrifuged at 3,000 *g* for 15 min. After subsequently discarding the supernatant, the precipitate was resuspended with 1–3ml PBS (pH = 6.8) for further culture and AFBS test.

### Microscopy Screening for AFBS

AFBS was prepared according to Ziehl–Neelsen method (Baso Diagnostics Inc., Zhuhai, China) (Xia et al., 2013), for further examination under light microscopy (Cat. No. CKX53; Olympus Corporation, Tokyo, Japan).

### MTB Culture

The MTB culture was done as reported previously (Dorman et al., 2018). In brief, 0.2 ml resuspended precipitate was inoculated on solid Lowenstein–Jensen (LJ) medium (Encode Medical Engineering Co., Ltd., Zhuhai, China) for 6–8 weeks. A total of 0.5 ml of the resuspended precipitate was also cultured on a liquid medium mycobacteria growth indicator tube (MGIT) 960 system (Becton, Dickinson and Company, New Jersey, USA). The growth detection time for MGIT tubes was recorded by BACTEC MGIT 960 system. LJ tubes were checked every other day for mycobacterial colonies. Culture positive of acid-fast bacilli was confirmed by *M. tuberculosis* complex by MPT64/MPB64 antigen detection or line probe assays. A culture-positive TB case was defined as a patient with at least one culture positive for TB.

### GeneXpert MTB/RIF

The GeneXpert MTB/RIF assay testing was performed according to the manufacturer**’**s instructions, Cepheid (Xpert MTB RIF kit insert, 2015), and previously report (Zhou et al., 2021). Briefly, the tissue was cut into small pieces and suspended with 2 ml PBS and then subsequently ground into a tissue homogenate using a tissue grinder. The 1-ml tissue homogenate was diluted with sample reagent at a ratio of 1:2 and shaken vigorously 10–20 times or vortexed for at least 10 s in a pretreatment tube. The mixture was incubated at room temperature for 15 min. Then, 2 ml of the resulting mixture was then transferred to the GeneXpert MTB/RIF cartridge and loaded into the machine, followed by the immediate operational procedure. All GeneXpert results were available within 2 h.2.7 T-SPOT assay

T-SPOT assays were performed according to the manufacturer’s instructions (Oxford Immunotec, Oxford, UK) (T-SPOT.TB ENGLISH package insert). In brief, peripheral blood mononuclear cells (PBMCs) were isolated and added to the 96-well plates precoated with anti-IFN-γ antibody. For each participant, medium well, PHA well, culture filtrate protein 10 (CFP-10) well, and early secreted antigenic target 6 (ESAT-6) well were prepared and tested. Plates were incubated at 37°C for 16–20 h. Anti-IFN-γ antibody conjugates and substrates were used to detect the presence of secreted IFN-γ. The number of distinct and dark blue spots were counted with an automated ELISPOT reader (CTL Analyzers, Cleveland, OH, USA). The test result is positive if (CFP-10-Nil) and/or (ESAT-6–Nil) ≥8 spots.

### TBAg/PHA Ratio

The ratio was calculated by dividing the positive spot numbers of ESAT-6 or CFP-10 by spot numbers of PHA. The larger of the two values was defined as the TBAg/PHA ratio of the patient.

### Statistical Analysis

The Mann–Whitney U-test and chi-squared test were used for non-parametric data between groups. An independent samples t-test was applied for quantitative data. Multivariable logistic regression analyses were performed with candidates from all tests with statistical significance. The prediction equation (diagnostic model) was developed by regression analysis, and a predictive score for every patient was calculated. The diagnostic accuracies of various models were determined through the receiver-operating characteristic (ROC) curve analysis. The area under the curve (AUC), specificity, sensitivity, predictive value, likelihood ratio, and accuracy, accompanied with their 95% confidence intervals (CI), were computed. Delong’s test between different models was computed through the R program (Version 4.1.2). Data analysis and graphs were performed using GraphPad Prism 7.0 and Statistical Package for Social Sciences software 26.0. All statistical tests were considered statistically significant when p < 0.05.

## Results

### Cohort Characteristics

A total of 519 patients were recruited and classified into two groups with spinal TB or non-TB (NTB). According to our diagnostic criteria, 203 patients were diagnosed with TB, while others were with NTB. In the Tongji Hospital cohort, there were 110 TB and 209 NTB. Another 200 patients were enrolled in Sino-French New City Hospital, including 93 spinal TB and 107 NTB. The clinical and demographic characteristics of our recruited individuals are presented in **Table 1**. There was no significant difference in age or sex between the two cohorts. The mean age was about 49 years old, and 62.62% of them were male.

**Table 1:** The clinical and demographic characteristics of recruited patients in two independent cohorts.

Variables	Tongji Hospital (training cohort)		P^$^	Sino-French New City Hospital (validation cohort)		P^$^	P^&^
	TB	NTB		TB	NTB		
Sex, male, %	72 (65.45%)	128 (61.19%)	0.460*	55 (59.14%)	70 (65.42%)	0.360*	0.964*
Age, years	49.20 ± 17.25	48.92 ± 17.78	0.881‡	48.68 ± 15.94	49.12 ± 14.22	0.835‡	0.949‡
Positive AFBS	17	1	p<0.001*	14	3	p=0.002*	0.206*
Positive T-SPOT	97	92	P<0.001*	83	48	p<0.001*	0.154*
TBAg/PHA ratio (median,95% range)	0.135 (0.007-2.218)	0.017 (0-0.201)	p<0.001†	0.115 (0.011-1.502)	0.009 (0-0.109)	p<0.001†	0.460†
Positive MTB culture	43	NA	p<0.001*	41	NA	p<0.001*	0.472*
Positive GeneXpert MTB/RIF	56	2	p<0.001*	59	3	p<0.001*	0.293*
Positive Histopathology	85	NA	p<0.001*	71	NA	p<0.001*	0.869*

TB, spinal tuberculosis; NTB, non-tuberculosis; AFBS, acid-fast bacilli smear; TBAg, Mycobacterium tuberculosis-specific antigen; PHA, phytohemagglutinin; MTB, Mycobacterium tuberculosis. NA, not available.

^$^Comparisons were conducted between TB and NTB.

^&^Comparisons were conducted between the training cohort and validation cohort.

^*^Chi-squared test.

^†^Mann–Whitney U-test.

^‡^Independent samples t-test.

### Results of Different TB Tests in Spinal TB and NTB

**Figure 2:**
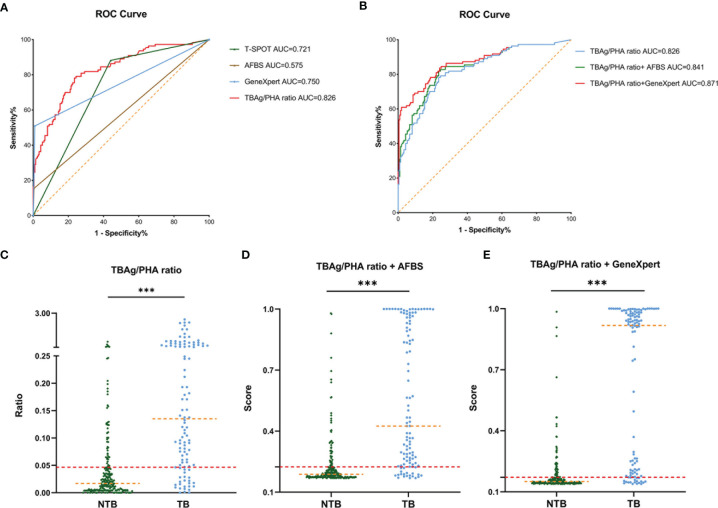
ROC curve of different tuberculosis tests and establishment of a diagnostic model in the Tongji Hospital cohort. **(A)** ROC analysis showing the performance of T-SPOT, AFBS, GeneXpert MTB/RIF, and TBAg/PHA ratio. **(B)** ROC analysis displaying models for the diagnosis of spinal tuberculosis based on the TBAg/PHA ratio or in combination with AFBS or GeneXpert MTB/RIF. **(C)** Scatter plots showing the ratio of TBAg/PHA in spinal tuberculosis patients (n = 110) and no spinal tuberculosis patients (n = 209). Orange dotted lines representing the median value. Red dotted lines indicating the cutoff value in distinguishing these two groups. ***p < 0.001 (Mann–Whitney U-test). **(D)** Scatter plots showing the score of the diagnostic model based on TBAg/PHA ratio combined with AFBS in spinal tuberculosis patients (n = 110) and no spinal tuberculosis patients (n = 209). Orange dotted lines representing the median. Red dotted lines indicating the cutoff value in distinguishing these two groups. ***p < 0.001 (Mann–Whitney U-test). **(E)** Scatter plots showing the score of the diagnostic model based on TBAg/PHA ratio combined with GeneXpert MTB/RIF in spinal tuberculosis patients (n = 110) and no spinal tuberculosis patients (n = 209). Orange dotted lines representing the median. Red dotted lines indicating the cutoff value in distinguishing these two groups. ***p < 0.001 (Mann–Whitney U-test). ROC, receiver operating characteristic; AUC, area under the curve; AFBS, acid-fast bacilli smear; MTB, *Mycobacterium tuberculosis*; RIF, rifampicin resistance; TBAg, *Mycobacterium tuberculosis*-specific antigen; PHA, phytohemagglutinin.

The ROC analysis was performed for each test individually. The AUC of AFBS, T-SPOT, TBAg/PHA ratio, and GeneXpert MTB/RIF was 0.575, 0.721, 0.826, and 0.750, respectively (**Figure 2A**). The lowest AUC was 0.575 obtained from AFBS. The best AUC was 0.826 obtained from TBAg/PHA ratio. The sensitivity and specificity of AFBS were 15.45% and 99.52%, respectively, while 88.18% and 55.98% for T-SPOT, respectively. The values of TBAg/PHA ratio sensitivity and specificity were 78.18% and 76.56%, respectively, when the best cutoff value was set at 0.047. The sensitivity and specificity of GeneXpert MTB/RIF were 50.91% and 99.04%, respectively. Thus, T-SPOT had better sensitivity, while AFBS and GeneXpert had better specificity, and the TBAg/PHA ratio was between the two. GeneXpert MTB/RIF had no statistical improvement in diagnosis compared to T-SPOT (p = 0.352, Delong’s test). However, the TBAg/PHA ratio showed better AUC than GeneXpert MTB/RIF (p = 0.008, Delong’s test) and T-SPOT (p < 0.001, Delong’s test). These results indicated that T-SPOT and TBAg/PHA ratio could be applied to distinguish the spinal TB and NTB, and TBAg/PHA ratio showed a better ability to distinguish spinal TB from NTB.

### Diagnostic Models Based on TBAg/PHA Ratio or Combined With Either AFBS or GeneXpert MTB/RIF

The specificity of AFBS and GeneXpert MTB/RIF was higher than TBAg/PHA ratio, but the sensitivity was the opposite. Since the AFBS and GeneXpert MTB/RIF were different from the T-SPOT method, their combination might help improve the AUC and accuracy of the diagnostic model. Thus, three diagnostic models were obtained by logistic regression analysis. The equation of TBAg/PHA ratio was P = 1/ [1 + e^-(-1.533 + 8.168 × TBAg/PHA ratio)^] (P, predictive value; e, natural logarithm). The diagnostic model of TBAg/PHA ratio combined with AFBS was P = 1/ [1 + e^-(-1.590 + 7.544 × TBAg/PHA ratio + 3.001 × AFBS)^] (P, predictive value; e, natural logarithm). The diagnostic model equation of TBAg/PHA ratio combined with GeneXpert MTB/RIF was P = 1/ [1 + e^-(-1.823 + 5.267 × TBAg/PHA ratio + 4.056 × GeneXpert MTB/RIF)^] (P, predictive value; e, natural logarithm). The AUC of TBAg/PHA ratio, TBAg/PHA ratio combined with AFBS, and TBAg/PHA ratio combined with GeneXpert MTB/RIF were 0.826, 0.841, and 0.871, respectively **(** **Table 2** and **Figure 2B**). The TBAg/PHA ratio combined with AFBS had an improvement in AUC than TBAg/PHA ratio (p = 0.023, Delong’s test), while TBAg/PHA ratio combined with GeneXpert MTB/RIF had a larger AUC than TBAg/PHA ratio combined with AFBS (p = 0.007, Delong’s test). The sensitivities were 78.18%, 81.82%, and 83.64%, and the specificities were 76.56%, 76.56%, and 76.08% when the cutoff values were set at 0.047, 0.225, and 0.171, respectively, for TBAg/PHA ratio, TBAg/PHA ratio combined with AFBS, and TBAg/PHA ratio combined with GeneXpert MTB/RIF **(** **Table 2** and **Figures 2C–E**). In addition, TBAg/PHA ratio combined with GeneXpert MTB/RIF had the best positive predictive value (PPV), negative predictive value (NPV), positive likelihood ratio (PLR), negative likelihood ratio (NLR), and accuracy (**Table 2**). Considering GeneXpert MTB/RIF was much more expensive than AFBS, we decided to retain these prediction models.

**Table 2:** The predicting performance of various diagnostic methods for distinguishing between TB and NTB in the training cohort (Tongji Hospital).

Variables	Cutoff value	AUC (95%CI)	Sensitivity (95%CI)	Specificity (95%CI)	PPV (95%CI)	NPV (95%CI)	PLR (95%CI)	NLR (95%CI)	Accuracy
Diagnostic model based on TBAg/PHA ratio	0.047	0.826 (0.777-0.874)	78.18% (69.58%-84.88%)	76.56% (70.36%-81.79%)	69.71% (59.50%-78.29%)	84.52% (80.71%-87.87%)	3.335 (2.864-3.821)	0.285 (0.215-0.372)	76.17%
Diagnostic model based on TBAg/PHA Ratio combined with AFBS	0.225	0.841 (0.794-0.888)	81.82% (73.58%-87.91%)	76.56% (70.36%-81.79%)	74.30% (64.15%-82.37%)	86.76% (82.81%-90.06%)	3.491 (2.966-4.041)	0.237 (0.172-0.323)	78.37%
Diagnostic model based on TBAg/PHA ratio combined with GeneXpert MTB/RIF	0.171	0.871 (0.827-0.916)	83.64% (75.61%-89.39%)	76.08% (69.86%-81.36%)	76.66% (66.57%-84.40%)	87.86% (83.85%-91.11%)	3.497 (2.966-4.056)	0.215 (0.152-0.300)	84.55%

TB, spinal tuberculosis; NTB, non-tuberculosis; AUC, the area under the curve; TBAg, Mycobacterium tuberculosis-specific antigen; PHA, phytohemagglutinin; AFBS, acid-fast bacilli smear; PPV, positive predictive value; NPV, negative predictive value; PLR, positive likelihood ratio; NLR, negative likelihood ratio; CI, confidence interval.

### Validation of the Diagnostic Models in the Sino-French New City Hospital

**Figure 3:**
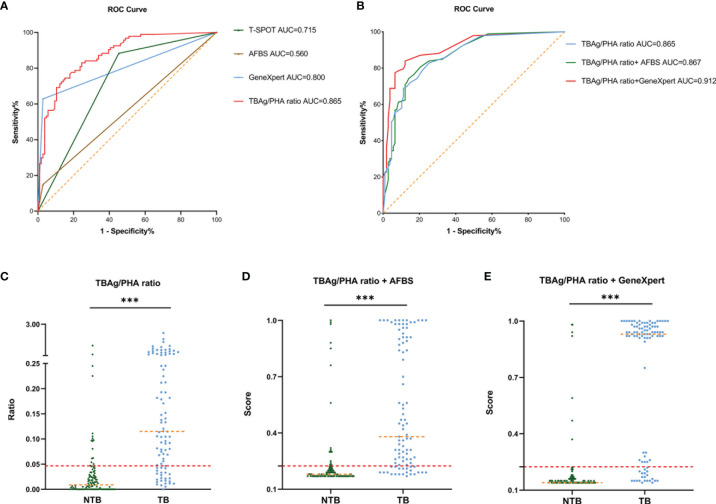
Validation of ROC curve of different tuberculosis tests and diagnostic models in the Sino-French New City Hospital cohort. **(A)** ROC analysis showing the performance of T-SPOT, AFBS, GeneXpert MTB/RIF, and TBAg/PHA ratio. **(B)** ROC analysis displaying models for the diagnosis of spinal tuberculosis based on the TBAg/PHA ratio or in combination with AFBS or GeneXpert MTB/RIF. **(C)** Scatter plots showing the ratio of TBAg/PHA in spinal tuberculosis patients (n = 93) and no spinal tuberculosis patients (n = 107). Orange dotted lines representing the median value. Red dotted lines indicating the cutoff value in distinguishing these two groups. ***p < 0.001 (Mann–Whitney U-test). **(D)** Scatter plots showing the score of the diagnostic model based on TBAg/PHA ratio combined with AFBS in spinal tuberculosis patients (n = 93) and no spinal tuberculosis patients (n = 107). Orange dotted lines representing the median. Red dotted lines indicating the cutoff value in distinguishing these two groups. ***p < 0.001 (Mann–Whitney U-test). **(E)** Scatter plots showing the score of the diagnostic model based on TBAg/PHA ratio combined with GeneXpert MTB/RIF in spinal tuberculosis patients (n = 93) and no spinal tuberculosis patients (n = 107). Orange dotted lines representing the median. Red dotted lines indicating the cutoff value in distinguishing these two groups. ***p < 0.001 (Mann–Whitney U-test). ROC, receiver operating characteristic; AUC, area under the curve; AFBS, acid-fast bacilli smear; MTB, *Mycobacterium tuberculosis*; RIF, rifampicin resistance; TBAg, *Mycobacterium tuberculosis*-specific antigen; PHA, phytohemagglutinin.

In an independent population in the Sino-French New City Hospital cohort, the blinded validation study was performed. There were similar results of different tuberculosis tests (**Figure 3A**). In the diagnostic model based on TBAg/PHA ratio, when the cutoff value of the validation cohort was set at 0.047, the AUC was 0.865, the sensitivity was 78.49%, and the specificity was 78.50%, which were obtained from the training cohort (**Table 3** and **Figures 3B, C**). In an analogous manner, with 0.225 as the threshold of the validation cohort, the sensitivity, specificity, and AUC of the diagnostic model based on TBAg/PHA ratio combined with AFBS were 82.26%, 77.11%, and 86.7%, respectively (**Table 3** and **Figures 3B, D**). After adding AFBS, there was no statistical improvement in AUC (p = 0.657, Delong’s test). When the cut-off value was 0.171, the same as the training cohort, the diagnostic model based on TBAg/PHA ratio combined with GeneXpert MTB/RIF performed well in the validation cohort, showing an AUC of 0.912 with 84.95% sensitivity and 85.05% specificity (**Table 3** and **Figures 3B, E**). The TBAg/PHA ratio combined with GeneXpert/MTB RIF had significant improvement in AUC compared to TBAg/PHA ratio alone (p = 0.0014, Delong’s test) or TBAg/PHA ratio combined with AFBS (p = 0.042, Delong’s test). Furthermore, TBAg/PHA ratio combined with GeneXpert MTB/RIF had the best PPV, NPV, PLR, NLR, and accuracy (**Table 3**).

**Table 3:** The predicting performance of various diagnostic methods for distinguishing between TB and NTB in the validation cohort (Sino-French New City Hospital).

Variables	Cutoff value	AUC (95%CI)	Sensitivity (95%CI)	Specificity (95%CI)	PPV (95%CI)	NPV (95%CI)	PLR (95%CI)	NLR (95%CI)	Accuracy
Diagnostic model based on TBAg/PHA Ratio	0.047	0.865 (0.815-0.915)	78.49% (69.10%-85.62%)	78.50% (69.81%-85.23%)	70.10% (58.96%-79.27%)	85.03% (81.11%-88.31%)	3.651 (2.836-4.678)	0.274 (0.206-0.363)	78.00%
Diagnostic model based on TBAg/PHA Ratio combined with AFBS	0.225	0.867 (0.817-0.917)	82.26% (73.28%-84.02%)	77.11% (68.31%-84.02%)	74.87% (63.79%-83.42%)	88.89% (85.47%-91.71%)	3.594 (2.798-4.586)	0.230 (0.166-0.318)	80.00%
Diagnostic model based on TBAg/PHA ratio combined with GeneXpert MTB/RIF	0.171	0.912 (0.872-0.953)	84.95% (76.30%-90.82%)	85.05% (77.08%-90.58%)	78.38% (67.41%-86.40%)	89.79% (85.61%-92.89%)	5.682 (3.962-8.100)	0.177 (0.119-0.262)	86.00%

TB, spinal tuberculosis; NTB, non-tuberculosis; AUC, the area under the curve; TBAg, Mycobacterium tuberculosis-specific antigen; PHA, phytohemagglutinin; AFBS, acid-fast bacilli smear; PPV, positive predictive value; NPV, negative predictive value; PLR, positive likelihood ratio; NLR, negative likelihood ratio; CI, confidence interval.

## Discussion

Spinal TB is the most common case of musculoskeletal TB, and specific evidence of culture is difficult to obtain due to the low culture rate of no more than 50% (Lan et al., 2013). The positive culture rate was 41.38% presented in our prospective study. Seriously, an accurate diagnosis of spinal TB takes an average of 1 year and 7 months (Sternbach, 1996). Although imaging (Marais et al., 2018; Jain et al., 2020), laboratory examination (Wang et al., 2019; Jain et al., 2020), histopathology (Thwaites et al., 2009; Jain et al., 2020), and other diagnostic methods have been studied, there is still no applicable diagnostic method to distinguish spinal TB rapidly and early from other diseases. In this study, we proved that the TBAg/PHA ratio showed a better capability to distinguish spinal TB from NTB, especially when used combined with GeneXpert MTB/RIF. To our knowledge, the present study is the first large-scale two-center prospective study embedded in routine clinical practice to assess T-SPOT assay and TBAg/PHA ratio in the evaluation of suspected spinal TB.

It is well-established that the early diagnosis is the basis of anti-spinal TB treatment. Some studies have shown that early control is required to prevent severe persistent kyphosis with attenuation of the spinal cord over the internal gibbus leading to paraplegia (Dunn and Ben Husien, 2018; Jain et al., 2020). As a hematological test, ELISA has developed to be a valuable test for active tuberculosis and latent tuberculosis infection for more than 10 years, whereas the ELISpot (T-SPOT) may be more sensitive (Lalvani and Pareek, 2010). CFP-10 and ESAT 6 are two early secretory proteins of *Mycobacterium* used in T-SPOT assay, showing their diagnostic ability in various tuberculosis including spinal TB (Kumar et al., 2010). The TBAg/PHA ratio is proposed to be more efficient in distinguishing TB from healthy people (Katakura et al., 2020; Wang et al., 2020). Luo et al. reported that TBAg/PHA ratio alone or combined with iron metabolism index presented a better capacity to distinguish active TB from latent TB (Luo et al., 2020b). Furthermore, Wang et al. also reported that TBAg/PHA ratio improved the diagnostic performance in differentiating EPTB (Wang et al., 2018). In consistent with the previous report, we proved that the diagnostic model of spinal TB based on the TBAg/PHA ratio in the T-SPOT assay had better efficacy, with the AUC value of 0.826 and 0.865 in the training and validation cohort, respectively. In addition, the TBAg/PHA ratio had also rebalanced the sensitivity and specificity of the T-SPOT assay, which might be interfered with the *Bacillus* Calmette–Guérin vaccine (Yan et al., 2015). Therefore, it suggests that the TBAg/PHA ratio can provide surgeons with preoperative diagnosis recommendations.

GeneXpert MTB/RIF is a rapid and effective PCR test for early spinal TB diagnosis (Patel et al., 2020). AFBS is a traditional and classical method through microscopy examination to diagnose TB in a short time (Vilchèze and Kremer, 2017). GeneXpert MTB/RIF was confirmed to be advantageous for TB diagnosis, particularly extra-pulmonary TB with negative AFBS (Mechal et al., 2019). Gerardo et al. reported that GeneXpert outperformed AFBS in all types of tuberculosis especially in cerebrospinal fluid (Alvarez-Uria et al., 2012). In this study, we found that GeneXpert MTB/RIF, with an AUC of 0.750, had a much better diagnostic efficiency than AFBS with an AUC of 0.575, in the training cohort. The infected bone tissues are superior to pus in the diagnosis of spinal TB, which is also confirmed in our TB culture results, consistent with Swapna report (Kanade et al., 2019). However, the positive rate of GeneXpert was <60% in our results because the diagnosis of spinal TB depends on the precise biopsy by the spine surgeon. Therefore, the diagnosis of spinal TB with GeneXpert MTB/RIF alone cannot meet the diagnostic requirements.

Since the T-SPOT is an assay based on immunity, we assumed that GeneXpert MTB/RIF could be a complementary method combination in the diagnosis. In our results, the TBAg/PHA ratio was proved to be superior to the T-SPOT results in diagnosing spinal TB. The diagnosis model based on the TBAg/PHA ratio combined with GeneXpert MTB/RIF significantly increased the AUC both in training and validation cohorts. The combination also had higher diagnostic PPV, NPV, PLR, NLR, and accuracy. Thus, the addition of GeneXpert MTB/RIF to the TBAg/PHA-based diagnostic model could improve the capacity to distinguish spinal TB from NTB. In other words, patients suspected of spinal TB need to undergo two tests, namely, the T-SPOT test and the GeneXpert MTB/RIF test. After the completion of the T-SPOT test, the predicted value of the TBAg/PHA ratio could be used for early warning of spinal TB. Then, a careful precise biopsy should be performed with skilled spine surgeons. If GeneXpert MTB/RIF results are negative, a second biopsy might be required when the TBAg/PHA ratio is much higher than the cutoff value. First, by calculating the prediction level of the combination of the TBAg/PHA ratio and GeneXpert MTB/RIF, it can guide early treatment when waiting for the gold standard diagnosis of spinal TB. For example, if a patient’s predictive value after the calculation is four times greater than the cutoff value (specificity >95%), combined with highly suspicious imaging, the patient is highly likely to have spinal TB. Then, preventive antituberculosis therapy is recommended before the gold standard diagnosis to alleviate symptoms, speed recovery, and prevent a postoperative recurrence. Second, the gold standard of EPTB has the problem of high missed diagnosis (Norbis et al., 2014), and preventive anti-tuberculosis treatment is sometimes required. Our model can provide preventive treatment suggestions for patients with the negative gold standard diagnosis but highly suspected spinal TB. As the diagnosis of spinal TB is complicated (Wang et al., 2019), our model also suffers from misdiagnosis and missed diagnosis. Thus, patients need to complete the gold standard test to provide conclusive evidence for subsequent standard treatment. In addition, our model has other shortcomings, which may be interfered with by latent TB. The diagnosis of latent TB is complicated. CT scan, clinical examination, medical history inquiry, and physical examination can be utilized to avoid the inclusion of latent TB. As a result, latent tuberculosis after exclusion has little effect on our research. How latent TB influences the outcome is an issue that needs further exploration in the future. GeneXpert machines are expensive and not easily accessible in some developing countries. Although the TBAg/PHA ratio combined with GeneXpert MTB/RIF had better performance than the TBAg/PHA ratio with AFBS in distinguishing spinal TB from NTB, the performance of the latter is better than that of TBAg/PHA alone. Considering the relatively simple technology and low price, the combination of TBAg/PHA ratio with AFBS could be also applied in underdeveloped areas or population.

It is widely known that spinal TB is most prevalent in developing countries and socioeconomically deprived communities. Other precise diagnostic methods may also be conducive to the individualized diagnosis of spinal TB. The real-time high-resolution imaging of TB colonies is a new technology that shortens the time to growth detection in culture (Ghodbane et al., 2015). TBDx system (Signature Mapping Medical Sciences, Herndon, USA) automatically loads slides onto a microscope and uses computerized algorithms to classify auramine-stained smears with a significantly higher sensitivity than manual microscopy (Lewis et al., 2012). Over 300 individual novel TB antigens including Rv2031c, Rv2029c, antigens of the Ag85 complex, and Rv0475 were reported in previous tests (Meier et al., 2018), providing other feasible strategies in the future. QuantiFERON-TB Gold (QFT) is also an IFN-γ release assay that might replace T-SPOT in the diagnosis of spinal TB (Acharya et al., 2020). Other nucleic acid probes and gene amplification techniques including loop−mediated isothermal amplification, line probe assay, or other gene-specific PCR (Acharya et al., 2020) could be used to investigate the diagnosis capacities of combined TBAg/PHA ratio. Studies of T-cell immune response (Jasenosky et al., 2015), subgroup immune cell changes (Cardona and Cardona, 2019), and genome sequencing (Huang et al., 2020) will be another way to distinguish spinal TB from NTB.

Notably, the present study had some issues to be addressed. First, to keep the independence of the training and validation cohorts, the sample size of the training cohort is relatively small. Second, the recruitment of patients was based on suspected spinal TB on radiological consideration. There existed missed diagnoses in radiologists’ reports and a bias between different radiologists. Third, since spinal TB is paucibacillary lesions, the fluoroscopy-guided percutaneous bone biopsy was limited by the disease itself. Although GeneXpert MTB/RIF relies on tissues from biopsy or operation, tissue dependence also occurs in MTB culture, AFBS, and histopathology. Fourth, although we try to avoid the inclusion of latent TB, it might interfere with the experimental results.

In conclusion, this study successfully provided generable evidence for the TBAg/PHA ratio of T-SPOT detection in distinguishing spinal TB from NTB in routine clinical application. The diagnostic model based on the combination of TBAg/PHA ratio with GeneXpert MTB/RIF possessed more effective results for spinal TB diagnosis.

## Data Availability Statement

The raw data supporting the conclusions of this article will be made available by the authors, without undue reservation.

## Ethics Statement

The studies involving human participants were reviewed and approved by Tongji Hospital, Tongji Medical College, Huazhong University of Science and Technology. The patients/participants provided their written informed consent to participate in this study.

## Author Contributions

YQ, YL, and FL co-designed the study. YQ wrote the first draft of the manuscript. XL, ZF, and YL wrote sections of the manuscript. YQ, ZL, ZF, YL, and FL performed research. YQ, ZL, and YL performed the statistical analysis. All authors contributed to the article and approved the submitted version.

## Conflict of Interest

The authors declare that the research was conducted in the absence of any commercial or financial relationships that could be construed as a potential conflict of interest.

## Publisher’s Note

All claims expressed in this article are solely those of the authors and do not necessarily represent those of their affiliated organizations, or those of the publisher, the editors and the reviewers. Any product that may be evaluated in this article, or claim that may be made by its manufacturer, is not guaranteed or endorsed by the publisher.

## Abbreviations

TB, tuberculosis; MTB, *Mycobacterium tuberculosis*; RIF, rifampicin; CRP, C-reactive protein; ESR, erythrocyte sedimentation rate; AFBS, acid-fast bacilli smear; IFN-γ, interferon-γ; EPTB, extrapulmonary tuberculosis; NALC, n-acetyl-l-cysteine; LJ, Lowenstein–Jensen; PBS, phosphate-buffered saline; PBMCs, peripheral blood mononuclear cells; CFP-10, culture filtrate protein 10; ESAT-6, early secreted antigenic target 6; ROC, receiver-operating characteristic; AUC, area under the curve; CI, confidence intervals; NTB, non-TB; PPV, positive predictive value; NPV, negative predictive value; PLR, positive likelihood ratio; NLR, negative likelihood ratio; QFT, QuantiFERON-TB Gold.
