# Supplementary material for: Tuberculosis-Specific Antigen/Phytohemagglutinin Ratio Combined With GeneXpert MTB/RIF for Early Diagnosis of Spinal Tuberculosis: A Prospective Cohort Study
Source: Front Cell Infect Microbiol. 2022 Jan 31;12:781315. doi: 10.3389/fcimb.2022.781315 (PMC8842995; doi:10.3389/fcimb.2022.781315)
Supplement: Supplementary file 1 [file DataSheet_1.pdf]

Supplementary Tables.

Table S1. Comparisons of AUCs between different TB tests by Delong's test

Model1	Model2	P-value (Delong's test)
T-SPOT	TBAg/PHA ratio	<0.001
T-SPOT	AFBS	<0.001
T-SPOT	GeneXpert MTB/RIF	0.352
TBAg/PHA ratio	AFBS	<0.001
TBAg/PHA ratio	GeneXpert MTB/RIF	0.008
AFBS	GeneXpert MTB/RIF	<0.001

Table S2. Comparisons of AUCs between different diagnostic models in the training cohort via Delong's test

Model1	Model2	P-value (Delong's test)
TBAg/PHA ratio	TBAg/PHA ratio combined with AFBs	0.023
TBAg/PHA ratio	TBAg/PHA ratio combined with GeneXpert	<0.001
TBAg/PHA ratio combined with AFBs	TBAg/PHA ratio combined with GeneXpert	0.007

Table S3. Comparisons of AUCs between different diagnostic models in the validation cohort by Delong's test

Model1	Model2	P-value (Delong's test)
TBAg/PHA ratio	TBAg/PHA ratio combined with AFBs	0.657
TBAg/PHA ratio	TBAg/PHA ratio combined with GeneXpert	0.001
TBAg/PHA ratio combined with AFBs	TBAg/PHA ratio combined with GeneXpert	0.004
